# Effect of sunitinib against *Echinococcus multilocularis* through inhibition of VEGFA-induced angiogenesis

**DOI:** 10.1186/s13071-023-05999-4

**Published:** 2023-11-07

**Authors:** Huijiao Jiang, Xiaoyi Wang, Lijiao Guo, Xiaowu Tan, Xianwei Gui, Zhenyu Liao, Zhiwei Li, Xueling Chen, Xiangwei Wu

**Affiliations:** 1 National Health Commission of the People’s Republic of China Key Laboratory of Prevention and Treatment of Central Asia High Incidence Diseases, First Affiliated Hospital, School of Medicine, Shihezi University, Shihezi, 832000 Xinjiang China; 2 Department of Immunology, School of Medicine, Shihezi University, Shihezi, 832000 Xinjiang China; 3 Department of Experimental Medicine, Jintang First People’s Hospital West China Hospital Sichuan University Jintang Hospital, Chengdu, 610400 Sichuan China

**Keywords:** *Echinococcus multilocularis*, Protoscolex, Metacestode tissue, Angiogenesis, SU11248, VEGFA, VEGFR2

## Abstract

**Background:**

Alveolar echinococcosis (AE) is a lethal zoonosis caused by the fox tapeworm *Echinococcus multilocularis.* The disease is difficult to treat, and an effective therapeutic drug is urgently needed. *Echinococcus multilocularis*-associated angiogenesis is required by the parasite for growth and metastasis; however, whether antiangiogenic therapy is effective for treating AE is unclear.

**Methods:**

The in vivo efficacy of sunitinib malate (SU11248) was evaluated in mice by secondary infection with *E. multilocularis*. Enzyme-linked immunosorbent assays (ELISAs) were used to evaluate treatment effects on serum IL-4 and vascular endothelial growth factor A (VEGFA) levels after SU11248 treatment. Gross morphological observations and immunohistochemical staining were used to evaluate the impact of SU11248 on angiogenesis and the expression of pro-angiogenic factors VEGFA and VEGF receptor 2 (VEGFR2) in the metacestode tissues. Furthermore, the anthelmintic effects of SU11248 were tested on *E. multilocularis* metacestodes in vitro. The effect of SU11248 on the expression of VEGFA, VEGFR2, and phosphorylated VEGFR2 (p-VEGFR2) in liver cells infected with protoscoleces in vitro was detected by western blotting, reverse transcription quantitative polymerase chain reaction (RT-qPCR), and enzyme-linked immunosorbent assay (ELISA). The influence of SU11248 on endothelial progenitor cell (EPC) proliferation and migration was determined using CCK8 and transwell assays.

**Results:**

In vivo, SU11248 treatment markedly reduced neovascular lesion formation and substantially inhibited *E. multilocularis* metacestode growth in mice. Further, it exhibited high anti-hydatid activity as efficiently as albendazole (ABZ), and the treatment resulted in reduced protoscolex development. In addition, VEGFA, VEGFR2, and p-VEGFR2 expression was significantly decreased in the metacestode tissues after SU11248 treatment. However, no effect of SU11248 on serum IL-4 levels was observed. In vitro, SU11248 exhibited some anthelmintic effects and damaged the cellular structure in the germinal layer of metacestodes at concentrations below those generally considered acceptable for treatment (0.12–0.5 μM). Western blotting, RT-qPCR, and ELISA showed that in co-cultured systems, only p-VEGFR2 levels tended to decrease with increasing SU11248 concentrations. Furthermore, SU11248 was less toxic to Reuber rat hepatoma (RH) cells and metacestodes than to EPCs, and 0.1 μM SU11248 completely inhibited EPC migration to the supernatants of liver cell and protoscolex co-cultures.

**Conclusions:**

SU11248 is a potential candidate drug for the treatment of AE, which predominantly inhibits parasite-induced angiogenesis. Host-targeted anti-angiogenesis treatment strategies constitute a new avenue for the treatment of AE.

**Graphical Abstract:**

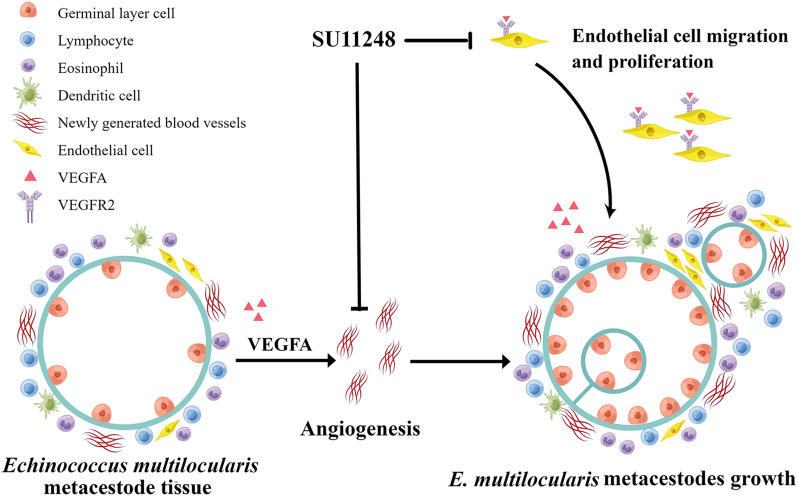

## Background

Alveolar echinococcosis (AE) is a severe life-threatening zoonotic parasitic disease caused by larvae of the fox-tapeworm *Echinococcus multilocularis* [1]. This affliction predominantly occurs in the cooler, temperate latitudes of the northern hemisphere and throughout Central and Eastern Europe, Central and Eastern Asia, and North America [2–5]. It is estimated that approximately 18,235 new cases of AE occur globally each year, of which 91% (16,629) are diagnosed in China, particularly in the Qinghai–Tibetan Plateau areas [6–8]. Human AE has an annual global impact of more than 687,823 disability-adjusted life years [9], which poses a significant economic and public health burden.

The life cycle of *E. multilocularis* depends on two mammalian hosts, with foxes and other canids as definitive hosts, while wild rodents are the natural intermediate hosts [10]. Humans are accidental intermediate hosts infected by ingesting parasite eggs [11]. After ingestion of eggs, the oncospheres released in the intestine are transported to the liver, where they typically settle and develop as larvae (termed metacestodes) [12]. The disease typically remains asymptomatic during the first 5–15 years. At diagnosis, most patients present with locally advanced or metastatic disease, so radical resection is not a viable option. Albendazole (ABZ) is an important antiparasitic drug for treating unresectable advanced human hydatid disease, and it is used as adjuvant therapy after surgery and in palliative treatment [13]. However, long-term ABZ administration can cause severe side effects and drug resistance [14]. Currently, few drugs are available for clinical treatment of AE; thus, novel alternative treatment options are urgently required.

The signaling pathways of parasites such as those that maintain the biological function of stem cells include bone morphogenetic proteins (BMPs)/BMP receptors (BMPRs)/small mothers against decapentaplegic proteins (Smads), Wnt/Frizzled/β-catenin, phosphatidylinositol 3-kinase (PI3K)/protein kinase B (PKB/AKT), and the fibroblast growth factor (FGF)/FGF receptor (FGFR) [15–18], or parasite-specific energy metabolic pathways that differ greatly from those of their hosts include the threonine degradation pathways [19, 20] and the malate mutation (MD) pathways [21–23], may be very promising therapeutic targets. However, AE is a rare and endemic parasitic disease, and the commercial rate of return on drug research and development is low. Even if the drug effect is excellent, it will require a long time for these new drugs to be successfully established in clinical practice. Therefore, it is more realistic to search among drugs that are already in the late development stage or are already available on the market.

In recent years, various anti-infective drugs [24–28], anti-cancer drugs [29–33], Chinese herbal medicines, and other drugs [34–37] have been examined in vivo and/or in vitro with regard to their anti-hydatid activity. However, none of these drug treatments successfully eliminated AE in the body, largely because the metabolic pathways of the parasite are similar to those of the host, and drug dosages that would be required to eliminate the parasite frequently cause substantial harm to the host [8, 38, 39]. In almost all cases, the host inevitably participates in drug metabolism. Therefore, rather than targeting the parasite with drugs, it is preferable to target the host directly. Successful parasitism resulting in *E. multilocularis* requires the establishment of a vasculature that communicates with the host, and the growth of the parasite has a mutually promoting effect on the generation of new blood vessels in its environment, which provide nutrients for the growth of the parasite and constitute a path for metastasis to distant sites [11, 40]. Regarding the elimination of the parasite, apart from the host immune system, the lack of blood supply is also an important cause of parasite death in vivo. Therefore, inhibition of angiogenesis may inhibit the development of AE.

Anti-angiogenic therapy has been widely used in the clinical treatment of various solid tumors [41], and it can produce significant improvements in ocular neovascular diseases [42], rheumatoid arthritis [43], and other diseases with abnormal angiogenesis. The vascular endothelial growth factor A (VEGFA)/VEGF receptor 2 (VEGFR2) signaling axis is one of the most important regulatory factors of angiogenesis. It participates in various physiological or pathological angiogenesis processes and is also the main target of anti-angiogenesis therapy [44]. Sunitinib malate (SU11248) is an orally administered multi-target tyrosine kinase inhibitor which has anti-angiogenesis and anti-tumor activity through targeted signal transduction of VEGFRs, and it has been approved for the treatment of advanced renal cell carcinoma [45–47].

Previous studies [48, 49] have confirmed that AE can lead to the continuous upregulation of the host VEGFA/VEGFR2 signaling pathway and constantly promote the formation of new blood vessels and the growth of new cysts. Here, we compare the activity of SU11248 and ABZ against AE in vivo with regard to the degree of angiogenesis and the expression levels of VEGFA, VEGFR2, and phosphorylated VEGFR2 (p-VEGFR2), to explore the effects of inhibiting angiogenesis on the development of AE. We co-cultured liver cells with protoscoleces to verify the effects of SU11248 treatment on various factors associated with angiogenesis at the cellular level, and we analyzed the effects of SU11248 on the migration and proliferation of endothelial progenitor cells (EPCs) through transwell and Cell Counting Kit-8 (CCK8) assays to further explore the anti-AE effects of SU11248 and the underlying mechanisms.

## Methods

### Study animals

Pathogen-free female C57BL/6 mice, aged 6–8 weeks and weighing 18–22 g, were purchased from the Laboratory Animal Center of Xinjiang Medical University and were raised in the animal facility of the College of Medicine, Shihezi University. Mongolian gerbils maintained in our laboratory were used for maintaining larval *E. multilocularis*.

### Cells and chemicals

Reuber rat hepatoma-35 (RH35) cells were purchased from Procell (Wuhan, China). Dulbecco’s modified Eagle medium (DMEM) and fetal bovine serum (FBS) were purchased from Gibco (Auckland, New Zealand). SU11248 and ABZ were purchased from APExBIO (Houston, TX, USA).

### Isolation of *E. multilocularis* protoscoleces

*Echinococcus multilocularis* protoscoleces were obtained from the infected Mongolian gerbils via intraperitoneal injection of minced metacestode tissue. After 4–6 months, the Mongolian gerbils were killed via CO_2_, and under fully sterile conditions, metacestodes were removed from the peritoneal cavity, sliced, and suspended in precooled phosphate-buffered saline (PBS) containing 1% penicillin/streptomycin. Following this, the protoscoleces were filtered into a 100-ml beaker using a 100-μm cell strainer and continuously washed with the precooled PBS until the filtrate supernatant was clear. These protoscoleces were used to inoculate the C57BL/6 mice intraperitoneally.

### Efficacy of SU11248 against *E. multilocularis* metacestodes in vivo

Female C57BL/6 mice (*n* = 18) were infected intraperitoneally with 2000 protoscoleces per individual. After 10 weeks, they were randomly assigned to three treatment groups of six individuals each. Treatments were carried out as follows: group 1 (control) received 0.2 ml honey/PBS solution (1:1 v/v) orally each day, group 2 (ABZ group) received 100 mg/kg ABZ in 0.2 ml honey/PBS solution (1:1 v/v) orally each day, and group 3 (SU11248 group) received 50 mg/kg SU11248 in 0.2 ml honey/PBS solution (1:1 v/v) orally each day. In addition, six uninfected female C57BL/6 mice were used as a blank control (group 4). After 6 weeks, the mice were killed using CO_2_ after eyeball blood was collected. Blood samples were centrifuged at 4000 rpm and 4 °C for 10 min to obtain serum. The serum was harvested and stored at −20 °C until interleukin (IL)-4, and VEGFA detection. The metacestodes in the abdominal cavity, the whole liver (containing metacestodes), and the spleen from each mouse were collected and weighed.

Several metacestodes from the abdominal cavity were fixed in 2.5% glutaraldehyde solution for scanning electron microscopy (SEM). The other metacestodes from the abdominal cavity and the liver, with or without hydatid cysts, were fixed in 4% paraformaldehyde for pathological examination.

### Efficacy of SU11248 against *E. multilocularis* metacestode vesicles in vitro

*Echinococcus multilocularis* metacestode vesicles were cultured as described previously, with some modifications [50]. The protoscoleces (1 ml) obtained from the previous procedure were co-cultured with 5 × 10^6^ RH35 feeder cells in 40 ml DMEM containing 10% FBS and 1% penicillin/streptomycin at 37 °C and 5% CO_2_. The medium was changed twice per week, and feeder cells were replaced weekly. After 4–6 weeks of culture, metacestode vesicles with a diameter of 2–5 mm were collected and were used for in vitro drug assays as described below.

To determine the cytotoxic effect of SU11248 on metacestode vesicles, 8–10 vesicles were cultured in each well of 24-well plates and were treated with various sunitinib concentrations (0, 0.04, 0.1, 0.5, 1, 10, 50, and 100 μM) daily for 7 days. Equal amounts of 40 μM ABZ and 0.1% DMSO were added as controls. All cultures were incubated at 37 °C under 5% CO_2_. Starting from the day of administration, the death of vesicles in each group was observed and recorded. Finally, structural changes in the germinal layer of metacestode vesicles per group were examined using SEM. Each experiment was carried out independently in triplicate for each group.

### Co-culturing mouse liver cells and protoscoleces

In vitro cultivation of the mouse liver cells was carried out as described previously [51]. Briefly, the mice were anesthetized through intraperitoneal injection with an anesthesia mix (3.75 ml/g body weight; final concentration of ketamine = 112.5 mg/kg and of xylazine = 22.5 mg/kg). The mice were placed on dissection trays, and limbs were secured. The inferior vena cava was cannulated, and the liver was perfused with warm perfusion buffer. After washing out blood and circulating cells, the liver was perfused three times (1 min/step). The liver was removed and placed in a 50-ml tube containing 20 ml of precooled 0.25 mg/ml Liberase (Merck KGaA, Darmstadt, Germany). After incubation for 30 min on ice, the capsule was cut, and the dissociated single cells were collected. The liver cells were subsequently centrifuged at 800 rpm for 20 min and resuspended in DMEM with 10% FBS. Viable cells were counted using trypan blue staining. The liver cells were plated on 60-mm plates at 10^5^ cells/plate, and the plates were then divided into six groups. Four of these groups were exposed to 2000 protoscoleces and the other two were used as the controls (liver cells only or protoscoleces only). The four co-culture groups were treated with SU11248 at final concentrations of 0, 40, 100, or 500 nM, respectively. All groups were incubated at 37 °C under 5% CO_2_ for 48 h. The harvested liver cells were centrifuged and were then collected to measure the expression of VEGFA, VEGFR2, and p-VEGFR2. The collected supernatants were used for measuring VEGFA levels using enzyme-linked immunosorbent assay (ELISA) and for transwell experiments.

### Assessment of in vitro toxicity in EPCs and RH cells

The migration, proliferation, and capillary tube formation of EPCs leads to neovascularization [52]. To evaluate the effects of SU11248 on EPC proliferation, cells were examined using a proliferation assay. Mouse bone marrow-derived EPCs were isolated, cultured, and identified as described previously [53]. EPC cells or RH35 cells were seeded in 96-well plates at a density of 1 × 10^4^ cells/well and were cultured overnight. The cells were then treated with the various concentrations (0, 0.04, 0.1, 0.5, 1, 5, 10, or 20 μM) of sunitinib for 24 h. Cell viability was measured using the Cell Counting Kit-8 (CCK-8, Biosharp, Shanghai, China) according to the manufacturer’s instructions. Median inhibitory concentration (IC_50_) values were calculated using the online IC50 Calculator tool (https://www.aatbio.com/tools/ic50-calculator) after logit-log transformation, and averages and standard deviations of six independent replicates were calculated.

### Transwell assay

To observe the effect of SU11248 on the migration of EPC cells, a transwell assay was used. After 48 h of serum starvation, EPCs (2 × 10^4^) were seeded into the uncoated upper chamber of 24-well plates (Corning, Inc., Corning, NY, USA) with 8-μm pore size for a migration assay. Cells in the upper chamber were cultured in medium with 1% FBS, with or without 40 nM SU11248, and the co-culture supernatants (500 μl) of the liver cells and protoscoleces were added to the lower chamber. As a negative control, untreated EPCs were added to the upper chamber, and the liver cell supernatants from the previous co-culture experiments were added to the lower chamber. After 16 h of culturing, cells on the bottom of the upper chamber were washed with PBS and fixed in 5% glutaraldehyde for 10 min. The cells were then stained with 0.1% crystal violet. Migrated cell populations were evaluated in five random fields per well at 200-fold magnification. Experiments were performed in triplicate.

### ELISA

VEGFA and IL-4 levels in the culture supernatants or the serum were determined using mouse VEGFA-specific or IL-4-specific ELISA kits (Cloud-Clone Corp., Wuhan, China), following the manufacturer’s instructions. Optical density was measured at 450 nm using a 96-well plate reader (Multiskan Ascent, Thermo Labsystems, Waltham, MA, USA), and the concentration was calculated according to a standard curve.

### Hematoxylin and eosin (HE) and immunohistochemistry (IHC) staining

HE staining was used to evaluate the effects of SU11248 on the pathological changes in metacestode tissue in mice. The tissue samples were removed and fixed in 4% paraformaldehyde at room temperature for 48 h, embedded in paraffin, and sectioned at 5-μm thickness. The slices were dewaxed and were stained with hematoxylin for 5 min and with eosin for 20 s. Thereafter, they were sealed with neutral resin and were examined using an optical microscope.

The sections were stained using the streptavidin–biotin–peroxidase method. After routine dewaxing and antigen reparation, the tissues were sealed with blocking buffer. Then, sections were incubated with the primary antibodies (dilutions: VEGFA, 1:500, VEGFR2, 1:200, p-VEGFR2, 1:50, and CD34, 1:3000, Abcam, Cambridge, UK), and results were visualized using a DAB chromogen device (Vector Laboratories, Burlingame, CA, USA), followed by counterstaining with hematoxylin.

Five visual fields in the inflammatory cell infiltration regions of metacestode tissues were analyzed in each section using an optical microscope (magnification: 200×), and 200 cells were counted per field. VEGFA/VEGFR expression was scored as follows: zero points, < 5% positive cells; one point, < 5–25% positive cells; two points, 26–50% positive cells; three points, 51–75% positive cells; and four points, > 75% positive cells. Staining intensity was rated as follows: one point, weak staining; two points, moderate intensity; and three points, strong intensity. Histological scores were calculated by multiplying the percentage and intensity scores, with results ranging from 0 to 12 [54, 55]. The staining was scored by two observers blinded with regard to sample information.

CD34 expression was visualized using a microscope to determine the microvessel density (MVD). A histological area (mm^2^) was considered, and MVD was calculated as reported previously [56]. Briefly, at 400-fold magnification, 10 optical fields were randomly chosen in the inflammatory cell infiltration regions of metacestode tissues, and microvessels were counted, with a single endothelial cell or cluster of endothelial cells considered one countable microvessel. The average MVD-CD34 counted in the 10 fields was converted and expressed as MVD/mm^2^.

### SEM

*Echinococcus multilocularis* metacestode vesicles or metacestode tissues were fixed in 2.5% glutaraldehyde buffer at 4 °C for 12 h and post-fixed in 2% solution of osmium tetroxide (OsO_4_) for 2 h at room temperature. The samples were washed three times with water, dehydrated with increasing concentrations of ethanol, and immersed in 2% isoamyl acetate for 20 h. The samples were then dried in a critical-point evaporator before sputter coating with gold for SEM.

### Western blotting

Total proteins were extracted from liver cells of different treatment groups using a Tissue Total Protein Extraction Kit (Sangon Biotech, Shanghai, China), and concentrations were determined using a BCA Protein Assay Kit (Sangon Biotech, Shanghai, China). Protein samples (20 µg) were separated by 10% sodium dodecyl sulfate polyacrylamide gel electrophoresis (SDS-PAGE) and transferred to a polyvinylidene difluoride (PVDF) membrane. The membranes were blocked in Tris-buffered saline plus Tween 20 (TBST) for 1 h at room temperature and were then incubated with specific primary antibodies against VEGFA (1:1,000, Abcam), VEGFR2 (1:1,000, Abcam), p-VEGFR2 (1:1,000, Abcam), and glyceraldehyde-3-phosphate dehydrogenase (GAPDH; 1:1,000, Cell Signaling Technology, Danvers, MA, USA) overnight at 4 °C. Thereafter, horseradish peroxidase (HRP)-conjugated goat anti-rabbit immunoglobulin G (IgG) secondary antibody (1:10,000, Zhongshan Goldenbridge Biotechnology, Beijing, China) was added for incubation at room temperature for 1 h. Blotted membranes were visualized using enhanced chemiluminescence reagents (Thermo Fisher Scientific, Waltham, MA, USA). Relative protein expression levels were quantified using ImageJ software and were normalized relative to GAPDH.

### Reverse transcription quantitative polymerase chain reaction (RT-qPCR)

Total RNA was isolated from the co-cultured liver cells with or without SU11248 treatment using TRIzol (Thermo Fisher Scientific). The purity and concentration of total RNA were measured using a NanoDrop 2000 spectrophotometer (Thermo Fisher Scientific) at 260/280 nm. Qualified RNA (2 μg) was reverse-transcribed into complementary DNA (cDNA) using a RevertAid First Strand cDNA Synthesis Kit (Thermo Fisher Scientific), which was sub-packaged and stored at −80 °C. The following PCR primers were used: VEGFA-F, 5′-TAGAGTACATCTTCAAGCCGTC-3′; VEGFA-R, 5′-CTTTCTTTGGTCTGCATTCACA-3′; VEGFR2-F, 5′-CTGGAGCCTACAAGTGCTCG-3′; VEGFR2-R, 5′-GAGGTTTGAAATCGACCCTCG-3′; β-actin-F, 5′-ACTGCTCTGGCTCCTAG CAC-3′; and β-actin-R, 5′-ACATCTGCTGGA AGGTGGAC-3′. The reaction buffers for RT-qPCR were prepared using a QuantiNova™ SYBR^®^ Green PCR Kit (QIAGEN, Düsseldorf, Germany), and RT-qPCR was conducted using a Bio-Rad CFX Manager system (Applied Biosystems, Foster City, CA, USA). The following cycling program was used: 95 °C for 15 min, followed by 40 cycles of 95 °C for 15 s and 60 °C for 30 s, and a final extension at 72 °C for 30 s. Fluorescence was measured at 72 °C. A melting curve analysis was performed after the final amplification period with a temperature gradient of 95 °C for 15 s, 60 °C for 15 s, and 95 °C for 15 s. The relative expression of target genes was calculated using the 2^−△△CT^ method [57]. Each experiment was carried out using three technical and three biological replicates.

### Statistical analysis

The data are presented as the mean ± standard deviation (SD) and were plotted with GraphPad Prism 8.0 software. Multiple comparisons between more than two groups were analyzed using a one-way ANOVA with Dunnett test or non-parametric Kruskal–Wallis test. A value of *P* < 0.05 was considered significant.

## Results

### Efficacy of SU11248 against *E. multilocularis* metacestodes in vitro

**Fig. 1:**
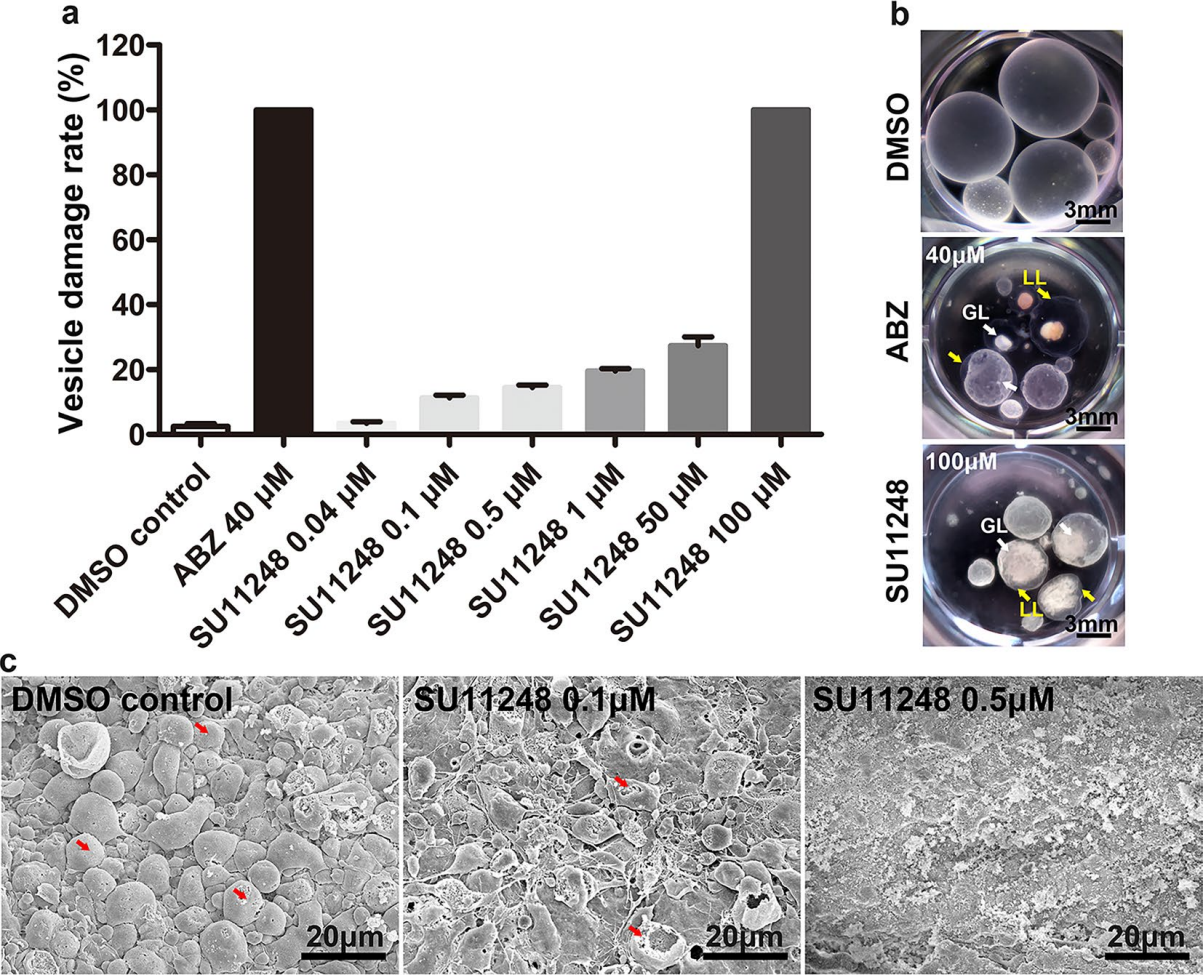
**a**–**c** Efficacy of SU11248 against *E. multilocularis* metacestode vesicles in vitro. **a** Axenically cultivated metacestode vesicles (eight per well in 2 ml volume) were incubated for 7 days in the presence of 0.1% DMSO or SU11248 at different concentrations (as indicated), and the structural integrity of the vesicles was monitored. The means ± SD of triplicate experiments are shown. **b** Representative gross photographs of metacestode vesicles after treatment with 0.1% DMSO, 40 μM ABZ, or 100 μM SU11248 for 7 days. The normal vesicles were round and full, with a smooth surface, and contained clear fluid. The dead vesicles exhibited loss of turgor, collapse of vesicles, detachment of the germinal layer (GL) from the laminated layer (LL), and formation of a densely packed aggregate inside the vesicles. Scale bar = 3 mm. **c** Morphological changes of germinal layers from metacestode vesicles detected by SEM after incubation with 0.1% DMSO (control) or SU11248 (0.1 μM,1 μM). The cells of the germinal layer (red arrows) were significantly reduced and damaged after treatment with SU11248. Scale bar = 20 μm

To examine whether SU11248 has a direct killing effect on metacestodes, we tested the effects of different concentrations of SU11248 on parasite survival in the metacestode vesicle culture systems. The addition of 0.04–100 μM SU11248 exhibited concentration-dependent effects on mature metacestode vesicle survival (Fig. 1a, b), which after cultivation for 7 days led to approximately 10–20% damaged vesicles in the presence of 0.1–1.0 μM SU11248, 30% damaged vesicles at 50 μM SU11248, and 100% at 100 μM SU11248. The damage to metacestodes as a result of SU11248 treatment was further demonstrated by SEM (Fig. 1c). After 0.5 μM SU11248 treatment, the major portion of the germinal layer in metacestodes was largely distorted, and only tissue residues remained; after treatment with 0.1 μM SU11248, the cells of the germinal layer were significantly reduced, and a part of them was damaged. By contrast, the solvent control of DMSO-treated metacestodes showed no significant damage to the germinal layer. These results indicate that the lower concentrations of SU11248 (0.5 μM) were sufficient for the metacestodes to lose their multicellular structures of the germinal layer, but higher concentrations of SU11248 (100 µM) were required to cause the massive individual metacestode death.

### Efficacy of SU11248 against *E. multilocularis* metacestodes in vivo

**Fig. 2:**
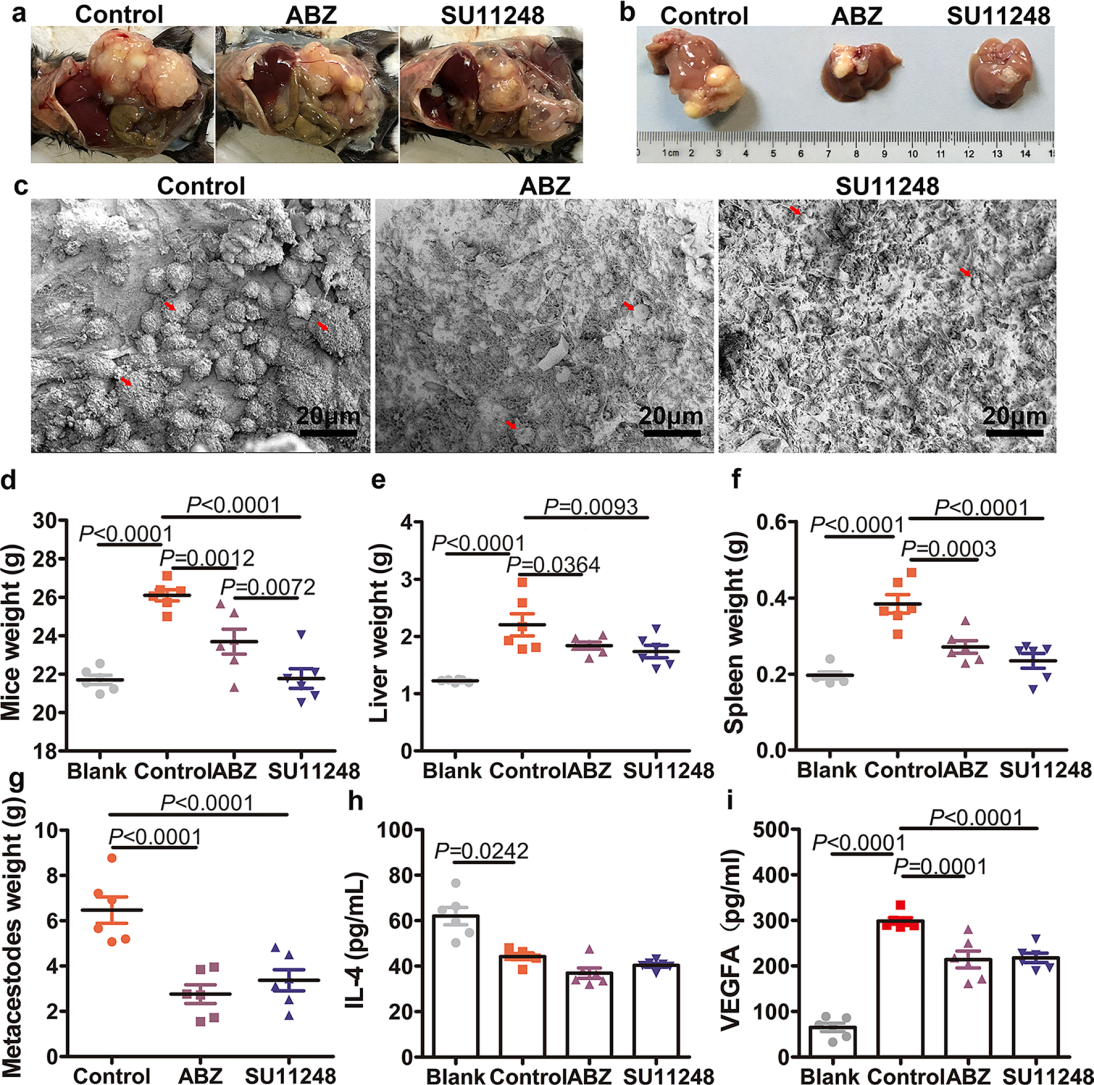
Efficacy of SU11248 against *E. multilocularis* metacestodes in vivo. Ten weeks after infection of C57BL/6 mice with protoscoleces, the mice were administered SU11248 intragastrically for 6 weeks. Uninfected, ABZ-treated, or PBS-treated mice served as the blank control, positive control, and negative control, respectively. **a** Gross morphology of metacestodes. **b** Intrahepatic manifestation for metacestode liver metastases in different treatment groups. **c** *Echinococcus multilocularis* metacestodes in different treatment groups were observed by scanning electron microscopy (SEM). The red arrow indicates the cells of the germinal layer. Scale bar = 20 μm. **d** Statistical analysis of mouse body weight changes. **e** Statistical analysis of liver weight changes. **f** Statistical analysis of spleen weight changes. **g** Statistical analysis of the intraperitoneal metacestode weight changes. **h**, **i** Cytokine expression in serum was measured by enzyme-linked immunosorbent assay. Serum levels of interleukin (IL)-4 (**h**) and vascular endothelial growth factor A (VEGFA) (**i**). The means ± SD are shown

Representative metacestode growth in mice of each treatment group after 6 weeks is indicated in Fig. 2a. In the control and ABZ groups, the hydatid cyst was solid, filled with yellow-white content, and surrounded by a layer of fibrovascular connective host tissue. In the SU11248 group, the hydatid cyst wall was thin and transparent, with few capillaries; the volume of the metacestodes was decreased, and most small vesicles in the cyst were filled with colorless fluid (Fig. 2a, b). SEM micrographs showed that the germinal layer of metacestodes from the SU11248 treatment group had lost their normal morphological characteristics, with cellular fragmentation and debris present (Fig. 2c). The wet weight of the extrahepatic intraperitoneal hydatid cyst in the SU11248 (2.76 ± 0.93 g) and ABZ groups (3.37 ± 1.05 g) was significantly lower than that in the control group (6.47 ± 1.31 g) (Fig. 2g). Similarly, the liver weight including metastatic metacestodes was lower in the SU11248 group (1.73 ± 0.24 g) than in the control group (2.21 ± 0.43 g); however, there was no significant difference in liver weight between the ABZ (1.84 ± 0.14 g) and SU11248 groups (Fig. 2e). The spleen weight in the SU11248 group (0.23 ± 0.04 g) was markedly lower than that in the control group (0.38 ± 0.05 g), which was similar to that of the ABZ group (0.27 ± 0.04 g) (Fig. 2f). Notably, the body weight of mice in the SU11248 group (21.78 ± 1.14 g) was significantly lower than that of mice in the control (26.11 ± 0.65 g) and ABZ (23.70 ± 1.46 g) groups (Fig. 1d). Compared to the control group, VEGFA levels were significantly decreased in the ABZ and SU11248 groups, while IL-4 levels were not significantly affected (Fig. 2h, i).

### SU11248 inhibition of *E. multilocularis* metacestode-induced angiogenesis

**Fig. 3:**
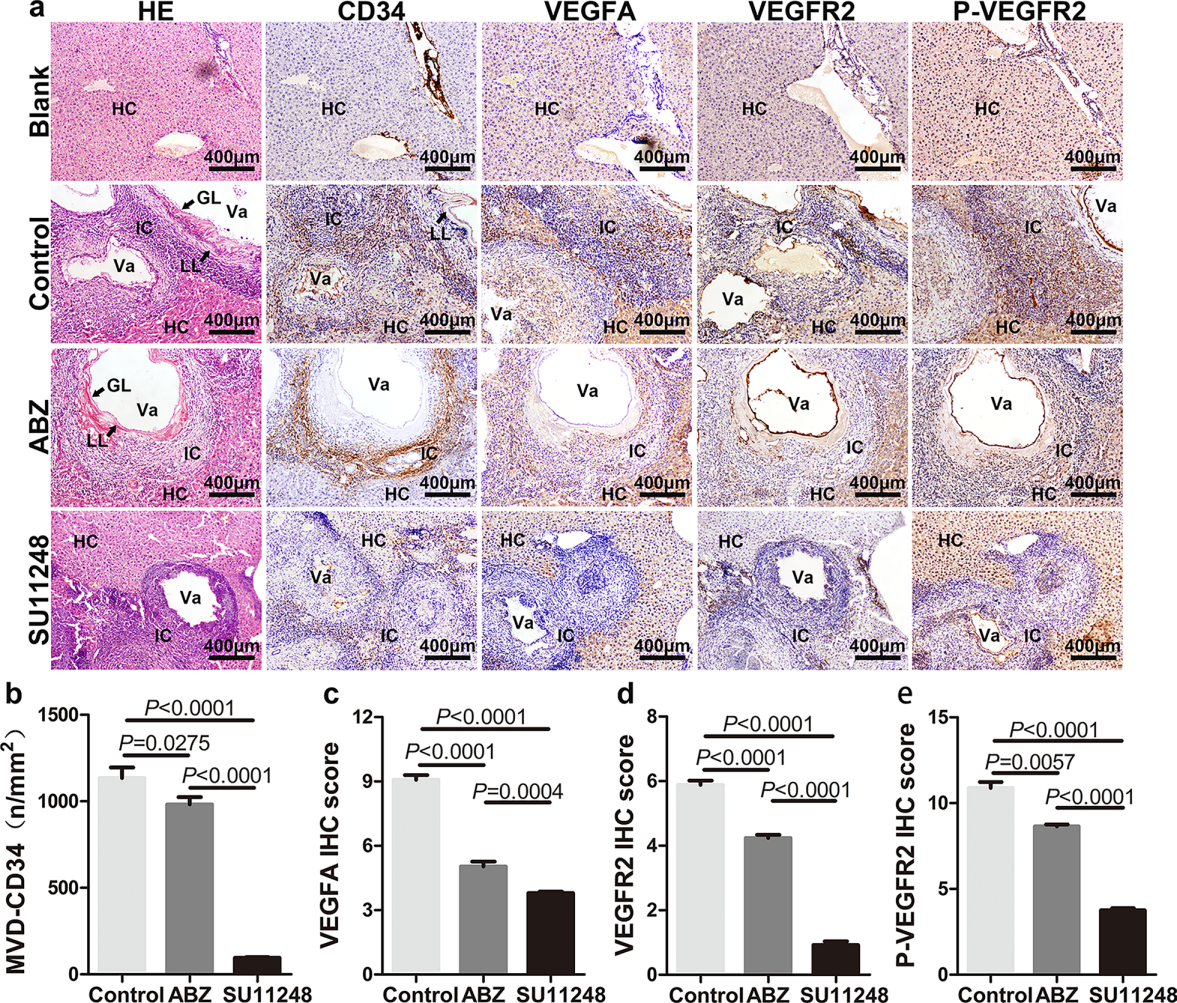
SU11248 inhibition of *E. multilocularis* metacestode-induced liver tissue angiogenesis. **a**–**e** Representative immunohistochemical staining and respective staining intensity scores. HE staining and immunohistochemical detection of the expression of four angiogenesis-related factors in liver metacestode tissues (**a**). PSC protoscolex, HC hepatic cell, IC inflammatory cell, Va vacuole, GL germinal layer, LL laminated layer. Microvessel density (MVD) determined by CD34 (**b**). Immunohistochemical scores of VEGFA (**c**), VEGFR2 (**d**), and p-VEGFR2 (**e**) in the four groups. Each group contains six samples. The means ± SD are shown

Angiogenesis is an important way for cestode *E. multilocularis* to capture nutrients from the host [16]. To further understand the effect of SU11248 on parasite growth and angiogenesis in vivo, the morphological changes in the metacestode tissues and the molecular regulation of angiogenesis were analyzed by HE and IHC staining. HE staining showed that in the control and ABZ groups, the hydatid cysts inside the liver were surrounded by numerous inflammatory cells, and intact germinal and laminated layers were observed (Fig. 3a). In contrast, after SU11248 treatment, the intrahepatic metacestode lesions showed granulomatous inflammation only, and few germinal and laminated layers were observed (Fig. 3a).

**Fig. 4:**
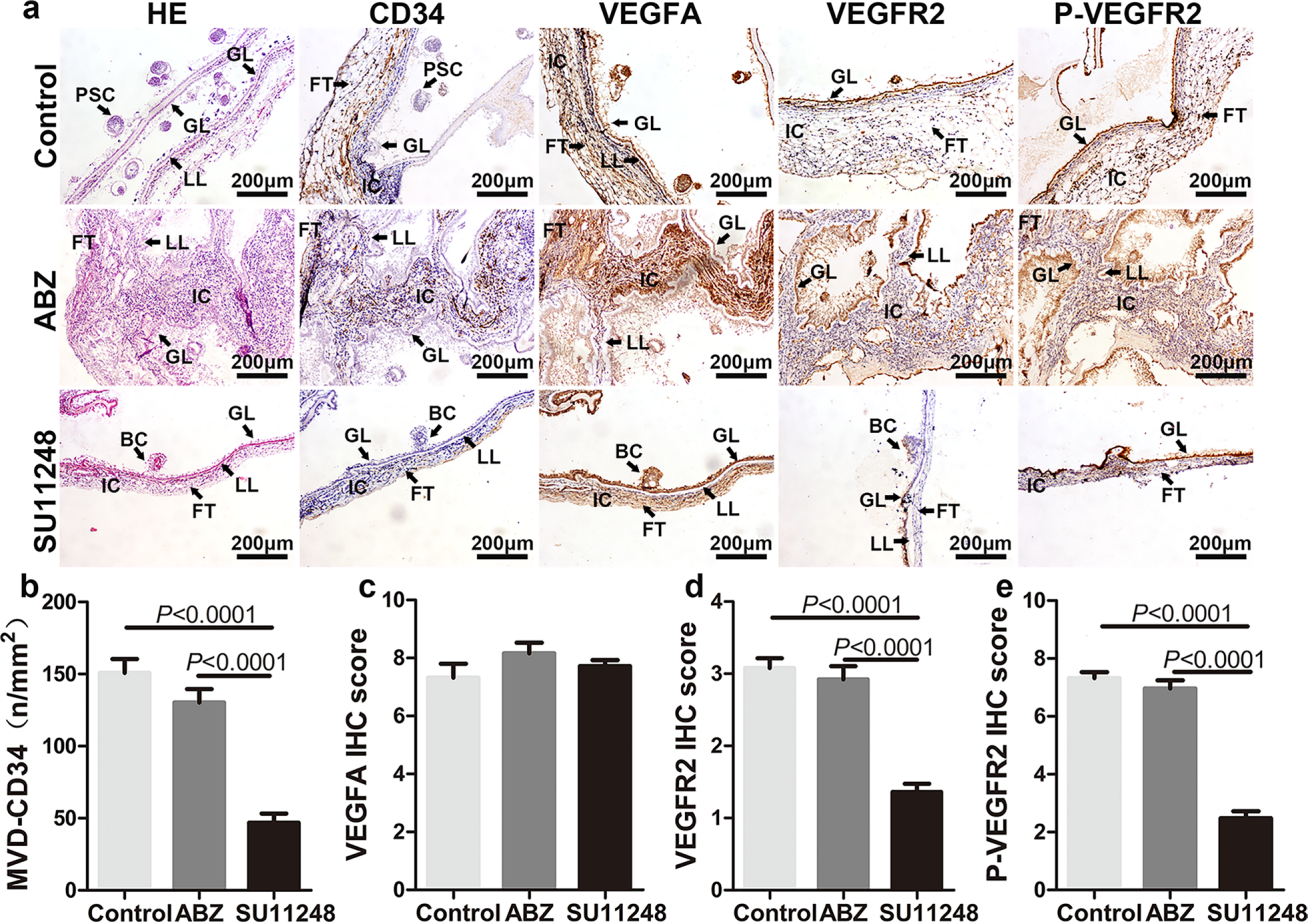
SU11248 inhibition of intraperitoneal *E. multilocularis* metacestode-induced angiogenesis. **a**–**e** Representative immunohistochemical staining and respective staining intensity scores. HE staining and immunohistochemical detection of the expression of four angiogenesis-related factors in intraperitoneal metacestode tissues (**a**). FT Fibrous connective tissue, (for other abbreviations, see the legend of Fig. 3). Microvessel density (MVD) determined by CD34 (**b**). Immunohistochemical scores of VEGFA (**c**), VEGFR2 (**d**), and p-VEGFR2 (**e**) in the three groups. Each group contains six samples. The means ± SD are shown

Pathological examination of the intraperitoneal hydatid cysts showed that the abundance of the protoscoleces was markedly reduced and their structure was altered in the ABZ and SU11248 treatment groups (Fig. 4a). The two drugs produced distinct effects on the fiber arrangement in connective tissues of the outer layer of metacestodes. In the ABZ group, the peripheral connective tissue of metacestodes was arranged in disorderly fashion, with light HE staining, and host cell infiltration was lower (Fig. 4a). In the SU111248 group, the connective tissue fibers were closely packed, the cells were compressed and deformed, and the nuclear volume was significantly reduced (Fig. 4a).

*Echinococcus multilocularis* metacestode-associated neovascularization was measured via microvessel density (MVD) via CD34 immunostaining. With regard to intrahepatic metacestodes, CD34 was expressed mainly in the epithelial cells of the granulomatous region (Fig. 3a), whereas among intraperitoneal metacestodes, CD34 was expressed mainly in peripheral connective tissue (Fig. 4a). In addition, microvessels inside the hydatid cysts were abundant, as confirmed by CD34 immunostaining, and they were distributed in the endophytic septum areas among the small daughter cysts (Fig. 4a). Compared to the control and ABZ groups, the MVD was significantly decreased in the hepatic (Fig. 3b) and the intraperitoneal metacestode tissues (Fig. 4b) after SU11248 treatment, indicating that the angiogenic response was severely attenuated.

### SU11248 inhibition of VEGFA, VEGFR2, and p-VEGFR2 expression in metacestode tissue

To understand the mechanism by which SU11248 suppresses metacestode-induced angiogenesis, we investigated the expression and distribution of angiogenesis-related proteins VEGFA, VEGFR2, and p-VEGFR2 in both the hepatic (Fig. 3) and the intraperitoneal metacestode tissues (Fig. 4). The majority of VEGFA and VEGFR2 expression occurred in the cytoplasm of the host inflammatory and endothelial cells, whereas p-VEGFR2 expression was mainly localized in the nuclei, in addition to lower expression in the cytoplasm (Figs. 3a, 4a).

Regarding hepatic metacestode tissues, the expression levels of VEGFA, VEGFR2, and p-VEGFR2 were significantly decreased in the SU11248 and ABZ groups, and they were lowest in the SU11248 group. Regarding intraperitoneal metacestode tissues, the expression levels of VEGFR2 and p-VEGFR2 were significantly decreased only in the SU11248 group, compared to the control group. However, there was no notable difference in VEGFA protein expression among the control, ABZ, and SU11248 groups. Together, these results clearly suggest that SU11248 treatment results in decreased VEGFR2 and p-VEGFR2 expression in metacestode tissues. However, whether SU11248 affects the expression of VEGFA requires further research.

### SU11248 inhibition of VEGFA-induced phosphorylation of VEGFR2 in mouse liver cells

**Fig. 5:**
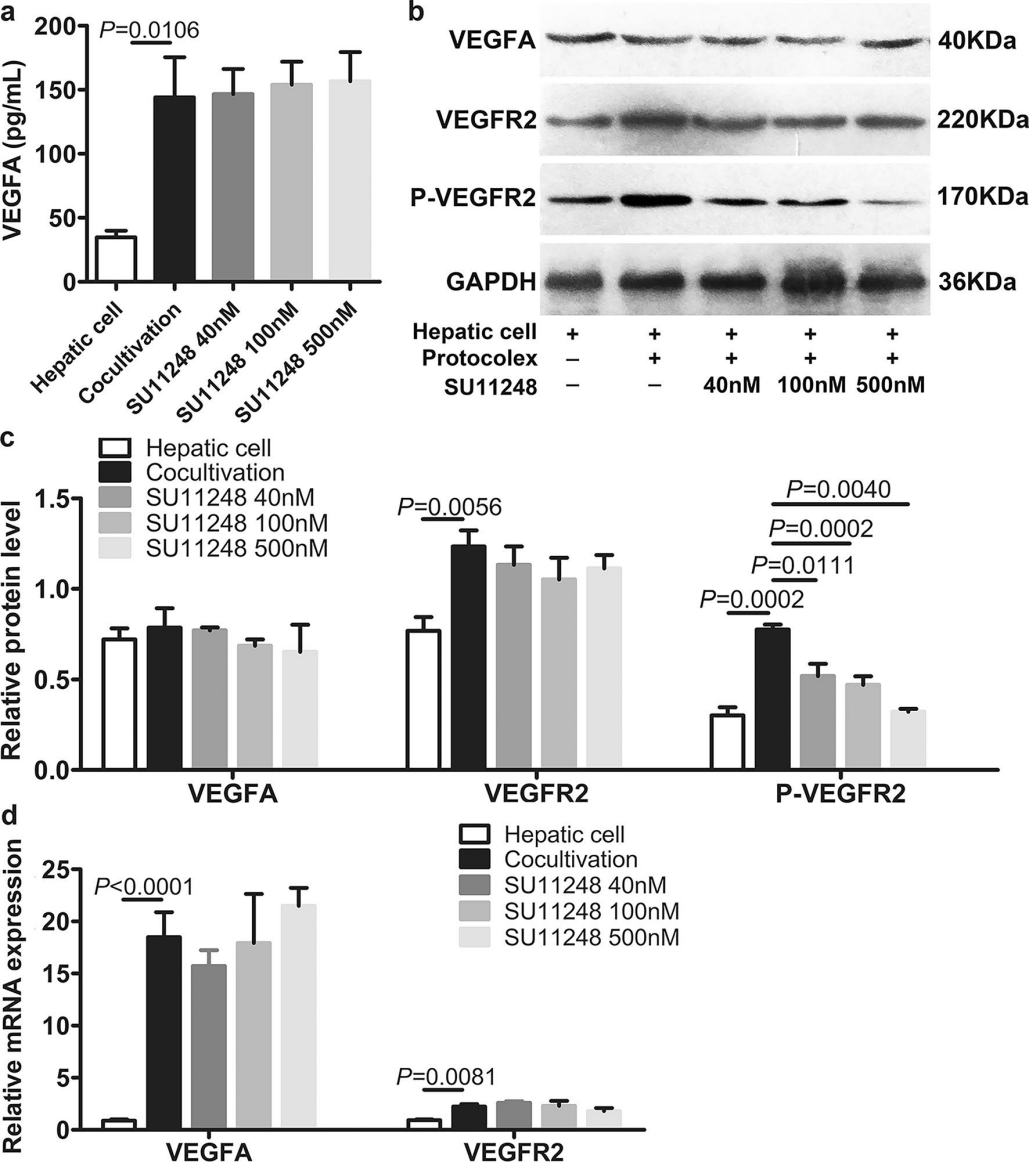
SU11248 inhibition of VEGFA-induced phosphorylation of VEGFR2 in liver cells from C57BL/6 mice. The hepatic cells were co-cultured with protoscoleces, and then SU11248 was added directly at final concentrations of 0, 40, 100, and 500 nM, with further incubation for another 48 h. The hepatic cell group co-cultured without protoscoleces was treated with 0.1% DMSO for 48 h. **a** Cell supernatants were analyzed for VEGFA by ELISA. The means ± SD of triplicate experiments are shown. **b** Western blotting was used to detect the protein expression of VEGFA, VEGFR2, and p-VEGFR2. **c** Quantitative analysis of VEGFA, VEGFR2, and p-VEGFR2 protein levels. The means ± SD of triplicate experiments are shown. **d** Messenger RNA expression levels of VEGFA and VEGFR2 were analyzed by reverse transcription quantitative polymerase chain reaction (RT-qPCR). The data are presented as the mean ± SD of triplicate experiments. For other abbreviations, see Fig. 1

To further verify the inhibitory effect of SU11248 on the expression of angiogenesis-related factors after parasite infection, we added different concentrations of SU11248 (0.04 μM, 0.1 μM, and 0.5 μM) into the liver cell/protoscolex co-culture system, and the expression of VEGFA, VEGFR2, and p-VEGFR2 was detected by RT-qPCR, western blotting, and ELISA. The ELISA showed that the levels of VEGFA in the media of the co-culture systems were higher than those in the media of control systems after 2 days of culturing; however, there were no significant differences among the co-culture systems with or without SU11248 (Fig. 5a). RT-qPCR demonstrated the same trend (Fig. 1d). Analysis of VEGFR2 expression (Fig. 5b–d) showed significant upregulation of VEGFR2 in co-cultured liver cells compared to the control cells. The VEGFR2 levels were decreased after adding various concentrations of SU11248, but without significant differences among them. As shown in Fig. 5b and c, a gradual decrease in p-VEGFR2 expression in co-cultured liver cells was observed after adding various concentrations of SU11248. These results suggest that co-culturing with protoscoleces induced higher expression of VEGFA, VEGFR2, and p-VEGFR2 in liver cells. In addition, SU11248 treatment did not directly downregulate VEGFA and VEGFR2 expression, and only p-VEGFR2 was downregulated.

### SU11248 suppresses mouse EPC proliferation and migration

**Fig. 6:**
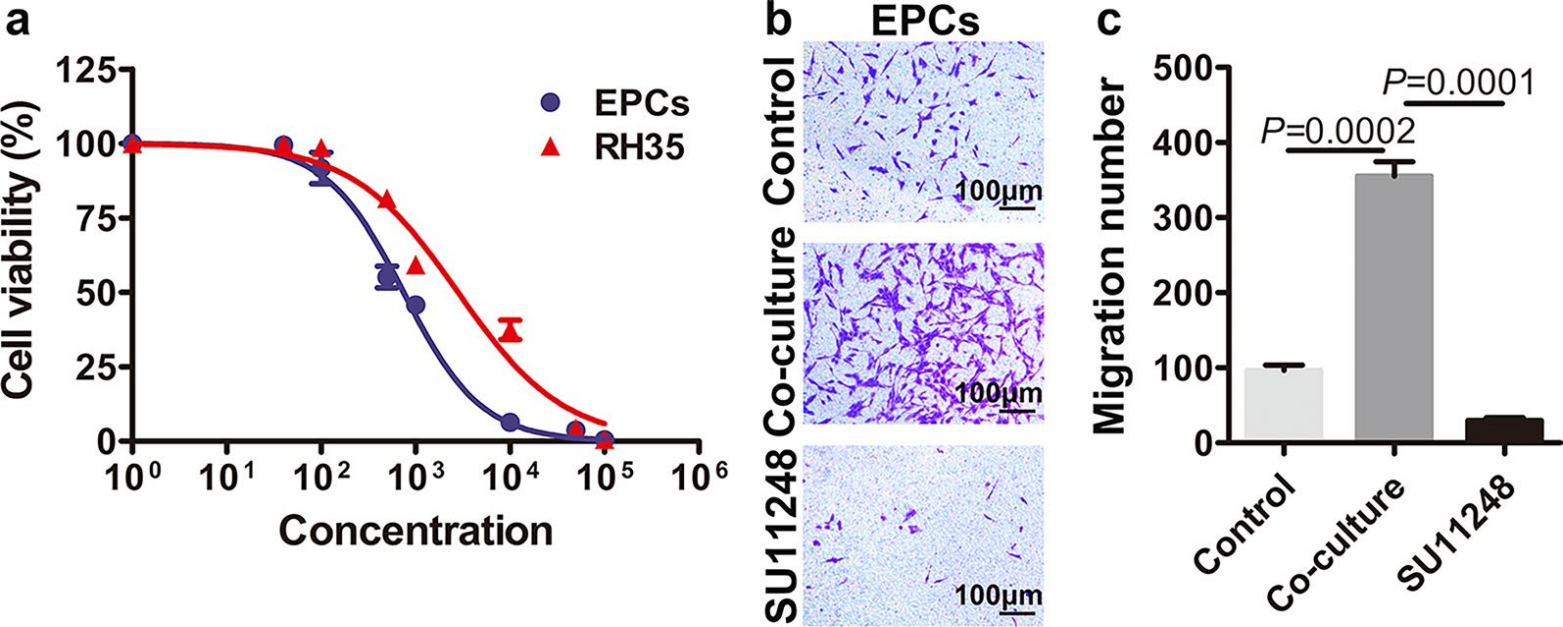
SU11248 suppresses mouse endothelial progenitor cell (EPC) proliferation and migration. **a** The effect of SU11248 on RH35 cell line proliferation and EPC proliferation was determined using CCK8 assays. IC_50_ values were calculated using the online IC50 Calculator. **b** The effects of SU11248 on the migration potential of EPCs were measured by transwell assay. The means ± SD of triplicate experiments are shown

To determine the cytotoxicity of SU11248 on RH35 cells and on EPC functions, we examined the effect of SU11248 on cell proliferation of mouse EPCs and RH35 cells using CCK-8 assay (Fig. 6a). The IC_50_ values of SU11248 in mouse EPCs were 0.75 ± 0.09 μM, and those of RH35 cells were 2.86 ± 0.46 μM; thus, EPCs were more sensitive to SU11248 than RH35 cells. Transwell assays showed that the media from liver cells and protoscoleces co-culture systems significantly enhanced the migratory ability of EPCs, whereas it was markedly inhibited by 0.1 μM SU11248 (Fig. 6b).

## Discussion

AE has certain biological characteristics that are similar to those of malignant tumors, such as infiltrative growth, intense neovascularization, distal metastasis, and recurrence. These characteristics may complicate treatment, resulting in a poor prognosis. Anti-hydatid drugs such as benzimidazole derivatives may not completely kill the parasite [14], they must be administered for long periods of time, and resistance may emerge [13]. Consequently, there is an urgent need to identify new drug targets and develop effective therapeutic strategies for AE. Infection with *E. multilocularis* metacestodes stimulates neo-angiogenesis, which promotes parasite proliferation and metastasis [40]. It is thus possible that suppressing angiogenesis may delay AE progression by interrupting the blood supply. In the present study, SU11248 treatment markedly reduced *E. multilocularis*-induced neovascularization. Meanwhile, it also inhibited protoscolex and nascent vesicle production. The treatment led to the formation of a thin fibrous wall of metacestode tissue, and parasite growth shifted from more individuals of small vesicles towards increased single-vesicle volumes. This might be because SU11248 inhibited neovascularization, resulting in a lack of nutrient supply to nascent vesicles and slow development, while growth continued in vesicles with adequate blood supply.

The effective range of total sunitinib concentration in the treatment of patients with metastatic renal cell carcinoma is 0.12–0.25 μM, whereas severe adverse reactions may occur at concentrations exceeding 0.25 μM [58, 59]. In our in vitro experiments, only approximately 20% of the metacestode vesicles were damaged after 7 days of continuous incubation with 0.5 μM SU11248, although SEM confirmed the disappearance of the cellular structure in the germinal layer of metacestodes in this treatment. Furthermore, the rate of vesicle damage was in the same range at 100 µM SU11248 as that at 40 μM ABZ. This concentration is well above the upper limit of the range acceptable for treatment. Thus, the immediate anti-hydatid effect of SU11248 is likely weaker than that of ABZ under an acceptable concentration of drug.

CD34 is the most sensitive endothelial marker, with the highest antigen specificity in nascent microvessels [60], and it is typically used to label microvessels and endothelial cells in neoangiogenesis activity studies. In our in vivo experiments, MVD-CD34 decreased by at least 68% in the SU11248 group compared with the control, whereas it decreased by at most 13% in the ABZ group. This confirmed that SU11248 significantly reduced the number of newly generated blood vessels at the perilesional tissues of metacestodes. Moreover, gross observations and HE staining supported this conclusion. Thus, we speculate that SU11248 exhibiting a similar effect to that of ABZ in vivo may be due to the inhibition of neovascularization.

Immunohistochemical analysis was performed to detect the expression of key angiogenesis-related molecules (VEGFA, VEGFR2, and p-VEGFR). As the *E. multilocularis* metacestode does not express the host CD34, VEGFA, and VEGFR2 proteins, we did not include the immunohistochemical analysis of the parasite structures in the germinal layer, brood capsule, and other parasites. *Echinococcus multilocularis* metacestodes can utilize the host nutrients for growth and development. The parasite can store host proteins for a prolonged duration. The alveolar hydatid cyst fluid also contains multiple host-derived proteins. This may be one of the reasons for the positive staining of various structures of the metacestodes in immunohistochemical analysis. After excluding the parasite staining interference, immunohistochemical analysis revealed that treatment with SU11248 significantly downregulated the expression of VEGFR2 and p-VEGFR2 in cells in the inflammatory response zone around the intraperitoneal and hepatic metacestodes. As VEGFA is a secreted factor, in addition to immunohistochemical staining, we performed ELISA to detect the peripheral blood levels of VEGFA in different groups of mice. SU11248 treatment downregulated the secretory levels of VEGFA in mouse peripheral blood. We then investigated the effects of SU11248 on liver cells after protoscolex infection in vitro and found that only the p-VEGFR2 level tended to decrease with increasing SU11248 concentration. This is concordant with the results of previous studies suggesting that p-VEGFR2 is a direct target of SU11248 [61, 62]. Further, SU11248 exhibited an apparent inhibitory effect on EPC proliferation and migration, indicating that EPCs may be effectors of the anti-angiogenesis action of SU11248.

Based on the above results, we speculated that in VEGFA-induced conditions in vivo, SU11248 specifically suppressed the p-VEGFR2 and VEGFA/VEGFR2 signaling pathways, leading to inhibited angiogenesis. Metacestode infiltrative growth was thus limited due to the lack of blood supply. At this time, the parasite was less able to invade new host tissues, resulting in reduced VEGFA and VEGFR2 expression and helping control the infection.

IL-4 is a pleiotropic Th2 lymphocyte-derived cytokine, and high IL-5 levels may contribute to the immune escape of *E. multilocularis* [63]. In our in vivo experiment, the serum IL-4 levels were significantly decreased in the control group, compared to the blank group, which was in line with the results of previous studies [34, 35, 64]; however, after treatment, the serum IL-4 content in the ABZ and SU11248 groups were not significantly different from those in the control groups, which differs from previous results [34, 35]. This discrepancy was probably because previous studies used BALB/c mice, which differ genetically from C57BL/6 mice, thus leading to different pathological immune responses.

An interesting finding from our experiment was the extensive death of cells in the germinal layer resulting from SU11248. This could be due to the inhibition of EmFGF signaling pathways by the tyrosine kinase inhibitor (SU11248), as it is important for parasite stem cell survival [18], which might be another mechanism of SU11248 against AE infection.

SU11248 can reverse immune suppression and modulation of the tumor microenvironment [65, 66], so it could be interesting to study the effects of combinations of SU11248 with other anti-hydatid drugs, including immunomodulatory activity of SU11248 or other anti-angiogenic drugs in the treatment of AE. In addition, several *E. multilocularis* proteins such as EmPGI may have a high pro-angiogenic potential and contribute to neovasculogenesis [67, 68]; thus, it may be of interest to identify the parasite proteins that elicit angiogenesis as well as their functioning.

## Conclusions

SU11248 is an AE treatment candidate that warrants further attention. The drug is commercially available and has been extensively characterized in terms of bioavailability, pharmacokinetics, and toxicity, which is favorable for rapid clinical use. We demonstrate here that the tyrosine kinase inhibitor SU11248 inhibits the development of AE by inhibiting the angiogenesis in vivo. This study identified anti-angiogenesis as a potential and promising drug target for the treatment of AE. In the future, more selective and less toxic antiangiogenic drugs may achieve better anti-AE medical efficacy.

## Data Availability

Data supporting the conclusions of this article are included in the article and its additional files.

## Abbreviations

ABZ: Albendazole
AE: Alveolar echinococcosis
cDNA: Complementary DNA
DMEM: Dulbecco’s modified Eagle medium
EPCs: Endothelial progenitor cells
ELISA: Enzyme-linked immunosorbent assay
FBS: Fetal bovine serum
GAPDH: Glyceraldehyde-3-phosphate dehydrogenase
HE: Hematoxylin–eosin
IC_50_: Median inhibitory concentration
IL: Interleukin
IHC: Immunohistochemistry
PBS: Phosphate-buffered saline
RH: Reuber rat hepatoma
RT-qPCR: Reverse transcription quantitative polymerase chain reaction
SEM: Scanning electron microscopy
SU11248: Sunitinib malate
SDS-PAGE: Sodium dodecyl sulfate polyacrylamide gel electrophoresis
VEGFA: Vascular endothelial growth factor A
VEGFR2: Vascular endothelial growth factor receptor 2
